# Acute and Chronic Toxicity, Cytochrome P450 Enzyme Inhibition, and hERG Channel Blockade Studies with a Polyherbal, Ayurvedic Formulation for Inflammation

**DOI:** 10.1155/2015/971982

**Published:** 2015-03-17

**Authors:** Debendranath Dey, Sunetra Chaskar, Nitin Athavale, Deepa Chitre

**Affiliations:** ^1^Bioved Pharmaceuticals Pvt. Ltd., BAIF Bhawan, Z Wing, Warje Malwadi, Pune 411 052, India; ^2^Bioved Pharmaceuticals, Inc., 1929 O'Toole Way, San Jose, CA 95131, USA

## Abstract

Ayurvedic plants are known for thousands of years to have anti-inflammatory and antiarthritic effect. We have recently shown that BV-9238, a proprietary formulation of *Withania somnifera, Boswellia serrata, Zingiber officinale,* and *Curcuma longa,* inhibits LPS-induced TNF-alpha and nitric oxide production from mouse macrophage and reduces inflammation in different animal models. To evaluate the safety parameters of BV-9238, we conducted a cytotoxicity study in RAW 264.7 cells (0.005–1 mg/mL) by MTT/formazan method, an acute single dose (2–10 g/kg bodyweight) toxicity study and a 180-day chronic study with 1 g and 2 g/kg bodyweight in Sprague Dawley rats. Some sedation, ptosis, and ataxia were observed for first 15–20 min in very high acute doses and hence not used for further chronic studies. At the end of 180 days, gross and histopathology, blood cell counts, liver and renal functions were all at normal levels. Further, a modest attempt was made to assess the effects of BV-9238 (0.5 *µ*g/mL) on six major human cytochrome P450 enzymes and ^3^H radioligand binding assay with human hERG receptors. BV-9238 did not show any significant inhibition of these enzymes at the tested dose. All these suggest that BV-9238 has potential as a safe and well tolerated anti-inflammatory formulation for future use.

## 1. Introduction

Modern medical pharmacotherapy is based on extensive testing and validation of efficacy and safety utilizing various methodologies. Toxicity manifestations of synthetic chemical drugs include hepatotoxicity, nephrotoxicity, neurotoxicity, and hematological, mutagenic, and cardiovascular toxicities [1, 2]. Complementary and Alternative Medicine (CAM) is rapidly becoming a viable option to modern pharmacotherapy for a multitude of chronic ailments. These include degenerative and inflammatory bone and joint conditions such as osteoarthritis and rheumatoid arthritis [3]. However, majority of these products have limited scientific evidence to validate their safety and efficacy [4]. Some of these supplements have shown significant side effects hence not considered to be safe even though they are natural in origin. A premarket approval and better reporting of safety of dietary supplements are also being promoted [5]. It is only in recent times that some of the single/multiple herbs have been tested for acute, subchronic, and chronic toxicity by modern testing methods [6–8]. Similarly, the traditional Ayurvedic science of medicine is known to use single or multiple plant combinations, especially for chronic conditions. These plant extracts usually consist of hundreds of active ingredients or molecules. Hence, evaluation of these multicomponent herbal products using conventional toxicological assessment methods is difficult. There is a need to apply modern methodologies to study and validate the safety of traditional herbs or plants and their combinations by a more extensive approach. This was a modest effort to study a formulation consisting of multiple standardized Ayurvedic plant extracts, for safety in in vitro and in vivo testing methods by modern pharmaceutical standards.

The test formulation, BV-9238, was developed based on concepts of the ancient science of Ayurveda [9–12]. Precursor formulations have been studied in controlled clinical studies in rheumatoid arthritis [13] and in osteoarthritis [14]. Recently the current authors have shown that it inhibits TNF-alpha (TNF-*α*) production from activated macrophages [15]. As is well known, the biologic TNF-*α* inhibitors have significant side effects such as life threatening infections and malignancies [16, 17]. This polyherbal formulation comprises specialized and proprietary extracts of four Ayurvedic medicinal plants, namely, Ashwagandha (*Withania somnifera*, Family Solanaceae), Shallaki (*Boswellia serrata*, Family Burseraceae), ginger (*Zingiber officinale*, Family Zingiberaceae), and Turmeric (*Curcuma longa*, Family Zingiberaceae). The doses and ratios of each plant were selected from a large spectrum of plants known for their anti-inflammatory and immunomodulatory properties. These four plants were selected due to their synergistic effects in inflammatory and degenerative conditions in different assays. Various dose ranging studies were conducted in in vitro, in vivo, and Phase-II clinical studies to arrive at the current dose and ratios of the four plants. A detailed description is beyond the scope of the current paper.

Most pharmaceutical drugs are metabolized by liver enzymes. Most patients are likely to be taking multiple drugs for various conditions. In such cases, a drug that is safe to be taken alone can easily become toxic when taken with one or more other drugs that are metabolized by the same liver enzyme/s. Cytochrome P450 (CYP450) is a family of isozymes responsible for the biotransformation of several drugs [18–20]. Drug metabolism via the CYP450 system has emerged as an important determinant in the occurrence of several drug interactions that can result in toxicity, altered bioavailability, increased or decreased pharmacological effect, adverse drug reactions, and/or drug-drug interactions [21]. Further, as dietary supplements are becoming more popular, people consume them in combination with Rx and OTC drugs and other supplements. Most of the times, toxicity profile of the supplements is not tested or not known; and there is a potential for drug-herb or even herb-herb interactions. There are several reports where a dietary supplement has elicited some drug-drug interactions and an initiative has to be taken to educate patients about the potential side effects of herbal supplements [5, 7, 22]. Going forward, it may be beneficial and preferable, or even mandatory, to document more detailed safety studies on supplement products. Our modest attempt was to demonstrate that a combination of 4 semipurified plant extracts can be tested along similar lines as Rx drugs.

Many drugs, especially in the treatment of inflammatory disorders such as arthritis, have a tendency to be associated with cardiotoxicity [23]. These drugs block cardiac ion channels which may lead to Torsades-de-Pointes (TdP) arrhythmia. A number of drugs have been withdrawn from the market and/or late stage clinical testing due to TdP risk [24, 25]. The rapid delayed rectifying potassium channel with a human isoform known as “human Ether-à-go-go-Related Gene” (hERG) plays an important role related to cardiotoxic effects [26]. Clinically, the ion channel proteins steer potassium ions out of the heart muscle cells, which affects the current in the cell membrane during the cardiac action potential.

In this study, we report the cell cytotoxicity and acute and chronic toxicity of BV-9238 in rats. Further, a modest attempt was made to address any future drug-drug interactions and any early signs of cardiac toxicity leading to QT prolongation or arrhythmias due to hERG channel inhibition.

## 2. Materials and Methods

### 2.1. Herbal Extracts

This polyherbal formulation comprises a combination of specialized and standardized extracts of the following Ayurvedic cultivated plants, namely,* Withania somnifera*,* Boswellia serrata*,* Curcuma longa*,* and Zingiber officinale*. The extraction method comprises a combination of water and organic solvent extractions, and each final extract is assayed for absence of residual solvents. The four herbs in BV-9238 were identified, authenticated, deposited, and registered at the Herbarium of Central Council for Research in Ayurvedic Sciences (CCRAS) in Pune, India. The herbarium voucher numbers are* Withania somnifera* (4390),* Boswellia serrata* (4391),* Zingiber officinale* (4393), and* Curcuma longa* (4392). The ratio of the four herbs in BV-9238 is as follows:* Withania somnifera (5)*,* Boswellia serrata (5)*,* Zingiber officinale (1.5)*,* and  Curcuma longa (1)*. Each of the four plants has been checked and is an accepted name by The Plant List (http://www.theplantlist.org/) Version 1.1 of September 2013.

**Figure 1:**
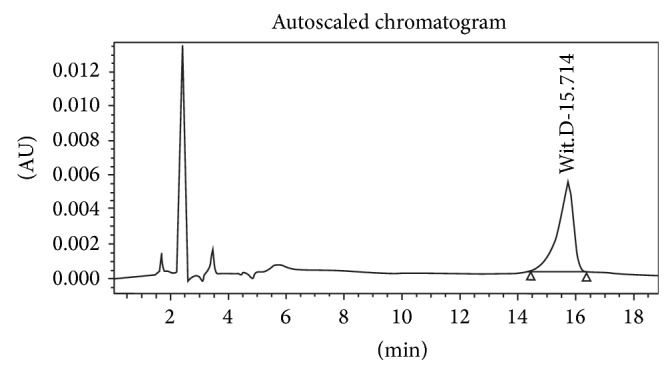
Standard Withanolide D.

**Figure 2 fig2:**
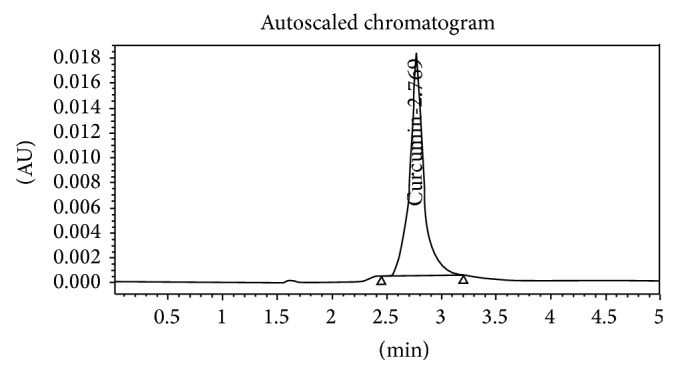
Standard curcumin.

**Figure 3 fig3:**
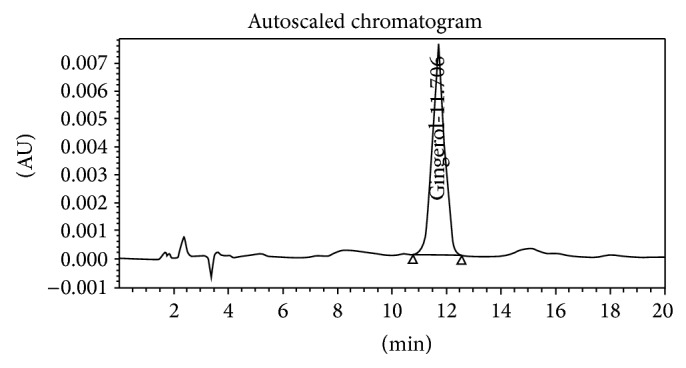
Standard gingerol.

**Figure 4 fig4:**
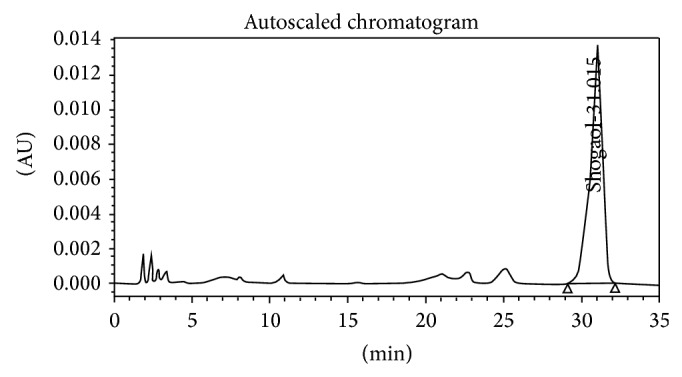
Standard shogaol.

**Figure 5:**
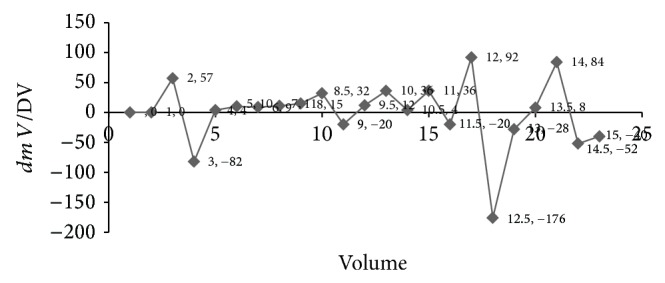
Total boswellic acids by potentiometry.

Each of the individual plants in BV-9238 and the final formulation were standardized using High Pressure Liquid Chromatography (HPLC), High Pressure Thin Layer Chromatography (HPTLC), and potentiometry [27]. The plants' corresponding standardization markers were obtained from well reputed research laboratories and Sigma Aldrich, USA, and are provided in parentheses,* Withania somnifera* (Withanolide D),* Boswellia serrata* (11-keto boswellic acid),* Curcuma longa* (curcumin),* and Zingiber officinale *(gingerol). For optimal efficacy, every single batch of plant extracts is ensured to the exact percentage of the standard markers. The sample chromatograms of each of the four plants are provided below in Figures 1 [Fig fig2] [Fig fig3] [Fig fig4]–5.

Five separate studies were conducted to evaluate the safety of the test formulation. Animal studies were carried out in compliance with Good Laboratory Practices (GLP) and test guidelines as defined in the European Community guidelines (EEC Directive of 1986; 86/609/EEC). The chronic toxicity studies were conducted per guidelines of The Organization of Economic Co-Operation and Development for testing of chemicals (OECD, 2002). Both acute and chronic toxicity studies were also designed and conducted per World Health Organization General Guidelines for Methodologies on Research and Evaluation of Traditional Medicine (WHO, 2000).

### 2.2. Cytotoxicity Assay

#### 2.2.1. Cell Culture

RAW 264.7 cells (ATCC, TIB 71, Rockville, MD, USA) were cultured in 75 cm^2^ plastic flasks (Corning-Costar) with Dulbecco's Modified Eagle's Medium (DMEM) and supplemented with 10% heat-inactivated Fetal Calf Serum (FCS3), penicillin (100 *μ*g/mL^−1^), and streptomycin (100 *μ*g/mL^−1^). For experiments, macrophages were detached by vigorous pipetting and centrifugation and plated in fresh medium. Cell viability was assessed by the mitochondria-dependent reduction of 3-(4,5-dimethylthiazol-2-yl)-2,5-diphenyltetrazolium bromide (MTT) to formazan. The cells were incubated with different doses (0.005–1 mg/mL) of BV-9238 in 96-well plates at 37°C for 24 h and with MTT (5 mg/mL^−1^) for 4 hours. The extent of reduction of MTT to formazan within cells was quantified (as indicator of total live cells) by measurement of OD_570_ against OD_630_.

### 2.3. Acute Toxicity

Six-week-old, male and female, Albino rats of Norwegian strain, weighing between 100 and 110 g, were used. After acclimation, animals were assigned to six treatment groups (5 rats/sex/group). They were housed under standard environmental conditions of temperature at 24 ± 1°C and 30–70% humidity under a 12-hour dark-light cycle and were allowed free access to drinking water and a balanced diet of standard animal feed. Animals were kept fasting, except for water, 16–18 hours prior to dosing. Doses of 2, 4, 6, 8, and 10 g/kg body weight (b.w.) of BV-9238 were given orally, in a single dose, to the different groups of rats, while the control group received only vehicle in the same volume by gavage. Body weight, signs of toxicity (general behavior, respiratory pattern, cardiovascular signs, motor activities, reflexes, and change in skin and fur), and mortality were observed after the drug administration at the end of the 1st, 2nd, 4th, and 6th hour and once daily till the end of the experiment (14 days). Weighed quantity of food (20 g) was made available to each cage. The leftovers were weighed and the quantity of food actually consumed was recorded. The differences were calculated, which were regarded as preliminary data of daily food consumption (g/animal/day).

### 2.4. Chronic Toxicity

Six-week-old, male and female, Albino rats of Norwegian strain weighing between 90 and 100 g were randomly divided into three groups (I, II, and III) of 40 rats (20 male and 20 female) each. Weighed quantity of food (20 g) was made available to each cage. For the study period, BV-9238 was administered orally at concentrations of 1 and 2 g/kg b.w. every day to the treatment groups for 180 consecutive days, while the control group received vehicle in the same dosage volume of treatment groups. These two doses were selected from the earlier single dose acute toxicity study, where we have barely seen any ataxia or ptosis. In order to assess reversibility, accumulation effects, or withdrawal, 20 rats (10 male and 10 female) from Group III were kept for 28 days posttreatment. In all groups, general behavior, respiratory pattern, cardiovascular signs, motor activities, reflexes, change in skin and fur, mortality, and body weight changes were monitored daily for the entire duration of the study [28]. The time of onset, intensity, and duration of these signs, if any, were recorded. At the end of the study, all animals were kept fasting for 16–18 hours and then were anesthetized on the 181st and 210th day (withdrawal groups). Blood samples for complete hemogram and full serum chemical analyses were taken in heparinized and normal blood sample tubes, respectively [29]. Urine samples of rats from all groups were subjected to qualitative analysis of nitrates, pH, protein, glucose, ketone, urobilinogen, and bilirubin.

All the animals were necropsied and gross pathological examination of all organs was performed. Weights were recorded of each of the following organs, namely, liver, kidney, heart, brain, spleen, adrenals, testes/ovaries, and lungs. All tissues were preserved in 10% neutral buffered formaldehyde solution. The tissues were embedded in paraffin and then sectioned and stained with haematoxylin and eosin and were examined microscopically.

### 2.5. Cytochrome P450 Enzyme Assay

Human recombinant CYP450 isomers (CYP1A2, CYP2C19, CYP2C9, CYP2D6, CYP2E1, and CYP3A4) expressed in* baculovirus* infected insect cells (BTI-TN-5B1-4) were obtained from BD Gentest (SUPERSOMES). The CYP450 enzyme assay for CYP1A2, CYP2C19, CYP2C9, CYP2D6, and CYP2E1 isomers employed 3-cyano-7-ethoxycoumarin as a substrate and for spectrofluorometric quantitation. CYP3A4 isomer used 7-benzyloxy-4-(trifluoromethyl)-coumarin as a substrate with spectrofluorometric quantitation of 7-hydroxy-4-(trifluoromethyl)-coumarin. The vehicle was 0.1% DMSO. BV-9238 at a dose of 0.5 *μ*g/mL and respective known inhibitors were at multiple doses to determine their IC50s. For this primary screen, the dose for BV-9238 was selected to translate around 10–12 *μ*M of active ingredients combined and with a goal that 50% of any inhibition would be noted as significant inhibition. The reaction incubation was carried out at 37°C in 75 mM potassium phosphate, pH 7.4 buffer, and stopped by stopping buffer as described [30, 31].

### 2.6. hERG Radioligand Binding Assay

Potassium channel hERG (human) was procured from HEK-293 which overexpressed human recombinant hERG (Eurofins Panlabs Inc.). The ligand was 1.5 nM [^3^H] Astemizole and the nonspecific ligand was 10 *μ*M Astemizole. The vehicle was 0.1% DMSO. BV-9238 was kept at 0.5 *μ*g/mL, which translates to 10–12 *μ*M of active ingredients combined. The experiment was carried out at 25°C for 60 minutes with the incubation buffer, 10 mM HEPES, pH 7.4, 0.1% BSA, 5 mM KCl, 0.8 mM MgCl, 130 mM NaCl, 1 mM EGTA, and 10 mM glucose. The *K*_*D*_ was 6.8 nM and *B*_MAX_ was 6.3 pmole/mg protein, with 90% specific binding [32, 33]. The goal was if it reaches significance (>50% inhibition) a further dose response curve will be undertaken.

The enzyme and radioligand binding assays were conducted by Eurofins Panlabs Inc., Bothell, Washington, USA. Methods employed were adapted from the scientific literature to maximize reliability and reproducibility. Reference standards were run as an integral part of each assay to ensure the validity of the results obtained. Biochemical assay results were presented as the percent inhibition of specific binding or activity using Spectrofluorimeter (Tecan Safire2, TECAN). The significance criterion was based on ≥50% inhibition at the tested dose and more doses were planned when it reached significance in tested criteria.

## 3. Results

### 3.1. Cytotoxicity Assay

As shown in Table 1, in mouse macrophage, RAW 264.7 cells, BV-9238 showed no toxicity at a wide range of doses from 0.005 to 0.5 mg/mL and 50% toxicity at 1 mg/mL after 24 hrs of treatment. In a subsequent study (data not shown) at higher dose, it did not affect any viability, most probably because of precipitation in cell culture plates. Control wells contained 0.1% DMSO as vehicle only and were considered as nondrug treated wells with 100% live cells. All these values are average of triplicate determinations.

### 3.2. Acute Toxicity in Rats

BV-9238 showed no mortality up to doses of 10 g/kg body weight when administered orally in rats. Further higher doses could not be administered due to experimental limitations of force feeding large amounts of test substance to the animals. All treated rats (males and females) showed anorexia up to 24 hours; thereafter the food consumption became normal. Body weight gain and food consumption overall of treated group were comparable to control group for the next 14 days (data not shown). Rats showed sedation, ataxia, and inhibition of motor activity and ptosis 15–20 minutes after administration of test formulation. The effects were found to be dose dependent, mild in the lower doses and marked in higher doses. The rats were found to be normal in all respects of behavior and food consumption beyond 24 hours posttreatment. No formulation related toxicity was seen in any rats, even at higher doses, after the first 24 hours till the end of the experiment.

### 3.3. Chronic Toxicity in Rats

There was no significant change in quantity of food consumption by control and treated animals. As a result, there was no significant change in the body weight gain of control and treated animals. The values of hematological parameters of treated animals and findings were comparable with that of control animals (Tables 2(a), 2(b), 2(c), and 2(d)). The values of biochemical parameters of treated animals were also comparable with that of control animals (Tables 3(a) and 3(b)). No abnormalities were noticed in urinalysis of treated animals and findings were comparable with that of control animals. This indicates the healthy status of liver and kidneys in the treated rats. An interesting thing is that a recent clinical study with another Ayurvedic formulation for osteoarthritis showed significantly elevated liver function tests in 30 weeks of study period [34]. In case of BV-9238, we did not see any increase in any of the liver enzymes in any of the sexes, even after 180 days of continuous treatment.

Gross pathology showed that the relative organ weights were comparable in control and treated animals. Necropsy and histopathological examinations were performed on the liver, kidneys, heart, brain, spleen, adrenals, testes/ovaries, and lungs of each animal to assess the damage to the internal organs or tissues. All the organs submitted essentially showed normal histology except in lungs and kidneys. 20 animals showed mild to severe bronchopneumonia, of which 2 were from the control group and 18 were from treated groups. Small whitish tubercles were noted on the lungs of 2 rats of vehicle control group, 3 rats of treated group (1 g/kg), and four rats of treated group (2 g/kg). This is attributed to force feeding of large amounts of test substance leading to aspiration pneumonia in the lungs. Also noted were edematous testes in 2 male rats of vehicle control group and in 1 male rat in each of the treated groups and mild to severe cloudy changes in the kidneys of all groups, 4/16 in control group and 5/16 in treated group that received 2 g/kg dosing. These changes were statistically nonsignificant.

Animals in the withdrawal study group showed no abnormal findings in body weight, food consumption, hematological and biochemical parameters, organ weights, and urinalysis and had similar findings on histopathology.

### 3.4. Cytochrome P450 Enzyme Assays

BV-9238 was examined for the ability to inhibit CYP450 enzymes, with the intent to determine which of the enzyme activities were most sensitive to inhibition (Table 4). CYP450 2C19 was most sensitive to inhibition, showing 35% inhibition of activity at a concentration of 0.5 *μ*g/mL of BV-9238 whereas CYP450 2E1, CYP450 2D6, and CYP450 2C9 activities were minimally sensitive. As reported in Material and Methods, none of these primary screens at single dose yielded any significant data to warrant a full-blown dose response study.

### 3.5. hERG Radioligand Binding Assay

This radioligand binding assay purely detects the affinity of the compound to its receptor. As shown in Table 5, positive control compound, Astemizole, showed an IC50 of 2.6 *μ*M whereas BV-9238 had no activity in the hERG radioligand binding assay. The negative value in this binding assay signifies that BV-9238 has no effect on the hERG channel or no potential cardiotoxicity leading to QT prolongation or arrhythmias as seen with some of the prescription drugs indicated in inflammation and bone and joint disorders.

## 4. Discussion

Nonsteroidal Anti-Inflammatory Drugs (NSAIDs) have long been the mainstay of treatment of all bone and joint, pain, and inflammatory conditions. The activity of NSAIDs is attributed to the inhibition of cyclo-oxygenase-1 enzyme (COX-1) and cyclo-oxygenase-2 enzymes. However, their long-term use is limited due to significant gastrointestinal side effects [35]. The selective cyclooxygenase-2 (COX-2) inhibitors were developed to protect against these gastrointestinal injuries [36, 37]. But COX-2 inhibitors, such as celecoxib, valdecoxib, etoricoxib, and parecoxib have a risk of cardiac arrhythmias and death. These agents can potentially cause fatal adverse cardiovascular events due to inhibition of the hERG potassium channel. This is the most critical channel for causing (TdP) arrhythmia and has become a critical component to assess cardiac safety of any new drugs [38, 39].

Herbal supplements are used commonly nowadays and often concomitantly with other prescriptions and over-the-counter drugs. As a result, there is major concern about potential harmful herb-drug interactions. The four plant extracts used in BV-9238 formulation have a long history of human use. Recent studies have reported their modulatory role in CYP450 systems, but only as individual plants. It was found that purified small molecules like 6- or 8-gingerols from ginger are much stronger inhibitors compared to whole ginger extracts [40]. In another study, cyclosporine bioavailability in animals went down only via oral delivery because ginger extracts blocked gastrointestinal mobility without any direct effect on CYP450 liver enzymes [41, 42]. Further, in human liver cells, it was demonstrated that pure 8- and 10-gingerols were inhibited, but 6-gingerols induced Cyp3A4 mRNA expressions [43].

Similarly it was found that total boswellic acids could be potent inhibitors, but other ingredients from* Boswellia serrata* extract play a key role as* Boswellia frereana* extract does not contain any of these boswellic acid forms [44]. Likewise, a pure curcumin molecule showed effect on cancer prevention by inhibiting the activation of carcinogens or aflatoxin-DNA adduct by modulating the CYP450 activity [45]. Also in mouse brain astrocytes, pure curcumin inhibits CYP450 2E1 upon oxidative stress as its antioxidant properties [46]. Different* Curcuma* species from the same family Zingiberaceae have been studied. It was found that two purified sesquiterpenes, zederone and germacrone, from* Curcuma elata* induced expression of CYP2B6 and CYP3A4 but not CYP1A2 mRNAs in human primary hepatocytes, thus providing some information on the safety of the* Curcuma* species [47].

*Withania somnifera* methanolic extract or pure molecules like Withaferin A, Withanolide A, or Withanoside IV did have some inhibitory activity of CYP3a only at very high dose (200 *μ*g/mL) in human and rat liver microsome study and did not alter significantly the pharmacokinetics of sildenafil citrate in rats [48]. In an interesting observation, Rana et al. showed that* Withania somnifera* may have some CYP450 reductase, a paralog enzyme for withanolide biosynthesis [49]. As mentioned before, our modest attempt, to see the four-plant combination effect on purified human cytochrome enzymes with only one dose, is very preliminary and exploratory. Keeping the plants in a semipurified and natural extract state may be safer than their purified chemical entities. A larger dose response study on CYP450 enzyme inhibition using several doses and their effect on induction of Phase-II enzymes, followed by an in vivo study, is needed to address these concerns.

In the current set of studies to assess safety, the acute toxicity study showed no mortality in doses up to 10 g/kg when administered orally to rats. Animals on study product showed a transient period of sedation, ataxia, and ptosis for less than 24 hours, which self-resolved. The rats were then found to be normal in all respects, such as behavior, body weight, and food consumption until the end of observation period. Larger doses could not be administered due to limitations in force feeding of the animals in study. Due to these transient findings, for the chronic toxicity study, it was decided to use doses of 1 and 2 g/kg b.w. for continuous treatment for 180 days.

The chronic toxicity study showed that the body weight gain and food consumption were comparable in both vehicle and treated groups. The values of hematological and biochemical parameters studied did not show any significant difference from the values of the control group. Of note is that the clotting time was similar in treated and control animals, signifying no toxic effect or risks with untoward bleeding or hemorrhagic events as seen with some of the NSAIDs [50]. The histopathological examination of all the organs was normal except in lungs and kidneys. It was found that few rats from all groups showed mild to moderate bronchopneumonia. These pathological changes can be attributed to long-term oral forced feeding resulting in mild aspiration of test substance into the lungs leading to bronchopneumonia. Also, few rats from all groups showed mild to severe cloudy changes in the kidneys. However, no abnormalities were seen in urinalysis of treated and control animals. These findings likely suggest that the study drug and vehicle were excreted through the kidneys and might affect proximal tubules leading to deposition of proteinaceous material in Bowman's space. There was no mortality in any of the groups.

Further, the test formulation does not have any adverse inhibitory effect on CYP450 and hERG assay. This suggests that the plant based formulation, BV-9238, does not have any significant potential for CYP450 interactions in oral treatment with other drugs. It also does not block the hERG channel, hence minimizing the risk of cardiac arrhythmias and death.

## 5. Conclusion

In these tests, this proprietary combination of standardized Ayurvedic plant extracts of* Withania somnifera*,* Boswellia serrata*,* Zingiber officinale*, and* Curcuma longa* was found to be safe and nontoxic. In vivo studies in rats suggest a very high margin of safety at doses in multiples of proposed therapeutic doses and no toxic effects on the major organs and laboratory hematological and chemistry testing measures in the study protocol. Further, in vitro studies, namely, CYP450 and hERG assay, suggest that the test product does not have any adverse hepatotoxic or cardiotoxic effects. Future studies may be considered to explore these pathways further.

## Figures and Tables

**Table 1:** Cytotoxicity of BV-9238 as determined by MTT assay.

BV-9238 concentration (mg/mL)	Well-1	Well-2	Well-3	Average (OD)
Control (0.1% DMSO)	2.301	2.066	2.064	2.143667
1	0.975	0.98	0.956	0.970333
0.5	1.974	2.091	2.027	2.030667
0.1	2.636	2.639	2.438	2.571
0.05	2.285	2.293	2.144	2.240667
0.025	2.368	2.299	2.275	2.314
0.01	2.111	2.134	2.099	2.114667
0.005	2	2.458	2.517	2.477667

**(a):** 

Dose	Group I	Group II	Group III	Group IV
	Vehicle control	1 g/kg/d	2 g/kg/d	2 g/kg/d
Hb (g%)	14.19 ± 0.40	14.19 ± 0.45^∗^	14.25 ± 0.09^∗^	13.75 ± 0.094^∗^
PCV (%)	36.79 ± 0.98	36.71 ± 1.04^∗^	35.85 ± 0.84^∗^	37.06 ± 0.728^∗^
RBC × 10^6^/cm	4.72 ± 0.05	4.62 ± 0.09^∗^	4.54 ± 0.07^∗^	4.55 ± 0.061^∗^
WBC total × 10^3^/cm	5.89 ± 0.17	5.63 ± 0.12^∗^	5.61 ± 0.11^∗^	5.58 ± 0.119^∗^
Clotting time (sec.)	154.50 ± 1.75	154.62 ± 0.72^∗^	153.88 ± 1.19^∗^	154.63 ± 1.13^∗^

**(b):** 

Dose	Group I	Group II	Group III	Group IV
	Vehicle control	1 g/kg/d	2 g/kg/d	2 g/kg/d
Neutrophils	46.00 ± 1.08	50.50 ± 0.38^∗^	50.10 ± 0.39^∗^	50.17 ± 0.42^∗^
Lymphocytes	52.00 ± 1.05	47.50 ± 0.46^∗^	48.06 ± 0.53^∗^	48.00 ± 0.42^∗^
Eosinophils	1.75 ± 0.25	1.73 ± 0.32^∗^	1.60 ± 0.17^∗^	1.60 ± 0.13^∗^
Monocytes	0.25 ± 0.14	0.28 ± 0.18^∗^	0.24 ± 1.19^∗^	0.23 ± 0.18^∗^

**(c):** 

Dose	Group I	Group II	Group III	Group IV
	Vehicle control	1 g/kg/d	2 g/kg/d	2 g/kg/d
Hb (g%)	14.00 ± 0.19	14.13 ± 0.10^∗^	14.23 ± 0.13^∗^	13.63 ± 0.156^∗^
PCV (%)	36.73 ± 0.96	36.36 ± 1.14^∗^	36.66 ± 0.84^∗^	37.57 ± 0.766^∗^
RBC × 10^6^/cm	4.59 ± 0.05	4.63 ± 0.08^∗^	4.58 ± 006^∗^	4.54 ± 0.062^∗^
WBC total × 10^3^/cm	5.72 ± 0.14	5.71 ± 0.07^∗^	5.64 ± 0.12^∗^	5.65 ± 0.105^∗^
Clotting time (sec.)	153.38 ± 1.21	155.13 ± 1.41^∗^	154.00 ± 1.07^∗^	154.87 ± 1.11^∗^

**(d):** 

Dose	Group I	Group II	Group III	Group IV
	Vehicle control	1 g/kg/d	2 g/kg/d	2 g/kg/d
Neutrophils	49.63 ± 1.10	50.13 ± 0.35^∗^	50.00 ± 0.50^∗^	50.15 ± 0.36^∗^
Lymphocytes	48.50 ± 0.13	48.15 ± 0.33^∗^	48.17 ± 0.48^∗^	48.14 ± 0.49^∗^
Eosinophils	1.63 ± 0.18	1.54 ± 0.18^∗^	1.54 ± 0.27^∗^	1.50 ± 0.00^∗^
Monocytes	0.24 ± 0.14	0.22 ± 0.18^∗^	0.29 ± 0.14^∗^	0.21 ± 0.18^∗^

Values are expressed as mean and standard deviation. ^∗^Values are nonsignificant when compared with Group I (control) animals.

**(a):** 

Dose	Group I	Group II	Group III	Group IV
	Vehicle control	1 g/kg/d	2 g/kg/d	2 g/kg/d
Total plasma protein (g%)	7.50 ± 0.12	7.60 ± 0.11^∗^	7.40 ± 0.11^∗^	7.45 ± 0.10^∗^
AST (IU/L)	48.80 ± 0.72	47.60 ± 1.00^∗^	46.70 ± 0.85^∗^	50.12 ± 0.95^∗^
ALT (IUL)	21.70 ± 0.72	22.80 ± 1.19^∗^	22.70 ± 0.84^∗^	21.21 ± 0.64^∗^
Blood urea (mg/dL)	20.30 ± 0.47	19.62 ± 0.39^∗^	19.20 ± 0.69^∗^	20.15 ± 0.39^∗^
Alk. phos. (IUL)	46.60 ± 1.21	45.40 ± 0.89^∗^	45.50 ± 0.87^∗^	44.56 ± 0.96^∗^
Blood glucose (mg/dL)	128.70 ± 1.63	128.05 ± 0.88^∗^	126.40 ± 0.94^∗^	125.98 ± 1.44^∗^
Cholesterol (mg/dL)	62.71 ± 0.91	62.99 ± 0.89^∗^	64.40 ± 1.21^∗^	64.10 ± 1.16^∗^
Triglycerides (mg/dL)	223.00 ± 3.46	220.50 ± 3.85^∗^	213.50 ± 2.31^∗^	215.00 ± 3.39^∗^

Values are expressed as mean and standard deviation. ^∗^Values are nonsignificant when compared with Group I (control) animals.

**(b):** 

Dose	Group I	Group II	Group III	Group IV
	Vehicle control	1 g/kg/d	2 g/kg/d	2 g/kg/d
Total plasma protein (g%)	7.50 ± 0.68	7.40 ± 0.12^∗^	7.40 ± 0.15^∗^	7.35 ± 0.10^∗^
AST (IUL)	50.20 ± 0.37	47.90 ± 1.85^∗^	48.82 ± 1.71^∗^	51.28 ± 1.26^∗^
ALT (IUL)	20.20 ± 0.57	22.40 ± 1.38^∗^	21.66 ± 1.12^∗^	21.94 ± 0.80^∗^
Blood urea (mg/dL)	20.50 ± 0.39	19.40 ± 0.58^∗^	18.50 ± 0.63^∗^	20.13 ± 0.39^∗^
Alk. phos. (IUL)	43.20 ± 0.20	46.02 ± 1.37^∗^	45.60 ± 0.88^∗^	44.95 ± 1.11^∗^
Blood glucose (mg/dL)	123.10 ± 0.76	128.60 ± 1.91^∗^	127.78 ± 1.76^∗^	125.46 ± 2.31^∗^
Cholesterol (mg/dL)	61.20 ± 0.73	62.90 ± 0.96^∗^	62.60 ± 1.07^∗^	63.04 ± 0.98^∗^
Triglycerides (mg/dL)	220.50 ± 3.35	215.00 ± 3.37^∗^	215.38 ± 1.93^∗^	216.62 ± 2.91^∗^

Values are expressed as mean and S.D. ^∗^Values are nonsignificant when compared with Group I (control) animals.

**Table 4:** Effect of BV-9238 at a dose of (0.5 *µ*g/mL) on six major isoforms of human CYP450 enzymes. Positive control inhibitors were tested in multiple doses and deduced IC50 (50% inhibitory concentration) is given in parenthesis.

	Enzymes tested	% inhibition	Control compound (IC50)
1	CYP450 1A2	4	Furafylline (1.07 *µ*M)
2	CYP450 2C19	35	Tranylcypromine (4.28 *µ*M)
3	CYP450 2C9	2	Sulfaphenazole (0.418 *µ*M)
4	CYP450 2D6	2	Quinidine (0.0345 *µ*M)
5	CYP450 2E1	1	4-Methylpyrazole (8.56 *µ*M)
6	CYP450 3A4	19	Ketoconazole (0.109 *µ*M)

**Table 5:** Effect of BV-9238 at a dose of (0.5 *µ*g/mL) on radioligand binding assay to potassium channel hERG receptors isolated from HEK-293 cells. Astemizole was kept as positive control and IC50 (50% inhibitory concentration) is given in parenthesis.

	hERG receptor	% inhibition	Reference compound (IC50)
1	Potassium channel hERG	−7	Astemizole (2.6 *µ*M)

## References

[1] Vora C. K., Mansoor G. A. (2005). Herbs and alternative therapies: relevance to hypertension and cardiovascular diseases. *Current Hypertension Reports*.

[2] Schoepfer A. M., Engel A., Fattinger K. (2007). Herbal does not mean innocuous: ten cases of severe hepatotoxicity associated with dietary supplements from Herbalife products. *Journal of Hepatology*.

[3] Michalsen A. (2013). The role of complementary and alternative medicine (CAM) in rheumatology—it's time for integrative medicine. *The Journal of Rheumatology*.

[4] Firenzuoli F., Gori L. (2007). Herbal medicine today: clinical and research issues. *Evidence-Based Complementary and Alternative Medicine*.

[5] Cohen P. A. (2014). Hazards of hindsight—monitoring the safety of nutritional supplements. *The New England Journal of Medicine*.

[6] Lahlou S., Israili Z. H., Lyoussi B. (2008). Acute and chronic toxicity of a lyophilised aqueous extract of *Tanacetum vulgare* leaves in rodents. *Journal of Ethnopharmacology*.

[7] Ha H., Lee J. K., Lee H. Y. (2010). Evaluation of safety of the herbal formula Ojeok-san: acute and sub-chronic toxicity studies in rats. *Journal of Ethnopharmacology*.

[8] Wanga M., Liub J., Zhoub B. (2012). Acute and sub-chronic toxicity studies of Danshen injection in Sprague-Dawley rats. *Journal of Ethnopharmacology*.

[9] Scartezzini P., Speroni E. (2000). Review on some plants of Indian traditional medicine with antioxidant activity. *Journal of Ethnopharmacology*.

[10] Thabrew M. I., Senaratna L., Samarawickrema N., Munasinghe C. (2001). Antioxidant potential of two polyherbal. Preparations used in Ayurveda for the treatment of rheumatoid arthritis. *Journal of Ethnopharmacology*.

[11] Chopra A., Patil J., Doiphode V., Patwardhan B. (2001). Exploring ancient Ayurveda for rheumatology; traditional therapy, modern relevance and challenges. *APLAR Journal of Rheumatology*.

[12] Dugasani S., Pichika M. R., Nadarajah V. D., Balijepalli M. K., Tandra S., Korlakunta J. N. (2010). Comparative antioxidant and anti-inflammatory effects of [6]-gingerol, [8]-gingerol, [10]-gingerol and [6]-shogaol. *Journal of Ethnopharmacology*.

[13] Chopra A., Lavin P., Patwardhan B., Chitre D. (2000). Randomized double blind trial of an Ayurvedic plant derived formulation for treatment of rheumatoid arthritis. *Journal of Rheumatology*.

[14] Chopra A., Lavin P., Patwardhan B., Chitre D. (2004). A 32-week randomized, placebo-controlled clinical evaluation of RA-11, an Ayurvedic drug, on osteoarthritis of the knees. *Journal of Clinical Rheumatology*.

[15] Dey D., Chaskar S., Athavale N., Chitre D. (2014). Inhibition of LPS-Induced TNF-*α* and NO production in mouse macrophage and inflammatory response in rat animal models by a novel ayurvedic formulation, BV-9238. *Phytotherapy Research*.

[16] Bongartz T. I., Sutton A. J., Sweeting M. J., Buchan I., Matteson E. L., Montori V. (2006). Anti-TNF antibody therapy in rheumatoid arthritis and the risk of serious infections and malignancies: systematic review and meta-analysis of rare harmful effects in randomized controlled trials. *The Journal of the American Medical Association*.

[17] Bernatsky S., Feldman D., De Civita M. (2010). Optimal care for rheumatoid arthritis: a focus group study. *Clinical Rheumatology*.

[18] Obach R. S. (2000). Inhibition of human cytochrome P450 enzymes by constituents of St. John's Wort, an herbal preparation used in the treatment of depression. *Journal of Pharmacology and Experimental Therapeutics*.

[19] Tang C., Shou M., Mei Q., Rushmore T. H., Rodrigues A. D. (2000). Major role of human liver microsomal cytochrome P450 2C9 (CYP2C9) in the oxidative metabolism of celecoxib, a novel cyclooxygenase-II inhibitor. *Journal of Pharmacology and Experimental Therapeutics*.

[20] Delgoda R., Westlake A. C. G. (2004). Herbal interactions involving cytochrome P450 enzymes. *Toxicological Reviews*.

[21] Zhou S., Gao Y., Jiang W., Huang M., Xu A., Paxton J. W. (2003). Interactions of herbs with cytochrome P450. *Drug Metabolism Reviews*.

[22] Ekor M. (2014). The growing use of herbal medicines: Issues relating to adverse reactions and challenges in monitoring safety. *Frontiers in Neurology*.

[23] Pratt C. M., Al-Khalidi H. R., Brum J. M. (2006). Cumulative experience of azimilide associated torsades de pointes ventricular tachycardia in the 19 clinical studies comprising the azimilide database. *Journal of the American College of Cardiology*.

[24] Darpo B. (2001). Spectrum of drugs prolonging QT interval and the incidence of torsades de pointes. *European Heart Journal*.

[25] Justo D., Prokhorov V., Heller K., Zeltser D. (2005). Torsade de pointes induced by psychotropic drugs and the prevalence of its risk factors. *Acta Psychiatrica Scandinavica*.

[26] Sanguinetti M. C., Tristani-Firouzi M. (2006). hERG potassium channels and cardiac arrhythmia. *Nature*.

[27] Chitre D., Dey D. (2010). Novel formulations to inhibit cyclooxygenase and pro-inflammatory cytokine mediated diseases. *US Patent Application Publication*.

[28] Chan P. K., O'Hara G. P., Hayes A. W., Hayes A. W. (1982). Principles and methods for acute and subchronic toxicity. *Principles and Methods of Toxicology*.

[29] Feldman B. F., Zinkl J. G., Jain N. C., Moor D. M. (2000). *Schalm's Veterinary Hematology*.

[30] Crespi C. L., Miller V. P., Penman B. W. (1997). Microtiter plate assays for inhibition of human, drug-metabolizing cytochromes P450. *Analytical Biochemistry*.

[31] Marks B. D., Smith R. W., Braun H. A. (2002). A high throughput screening assay to screen for CYP2E1 metabolism and inhibition using a fluorogenic vivid p450 substrate. *ASSAY and Drug Development Technologies*.

[32] Zhou Z., Gong Q., Ye B. (1998). Properties of HERG channels stably expressed in HEK 293 cells studied at physiological temperature. *Biophysical Journal*.

[33] Finlayson K., Turnbull L., January C. T., Sharkey J., Kelly J. S. (2001). [^3^H]dofetilide binding to HERG transfected membranes: a potential high throughput preclinical screen. *European Journal of Pharmacology*.

[34] Chopra A., Saluja M., Tillu G. (2013). Ayurvedic medicine offers a good alternative to glucosamine and celecoxib in the treatment of symptomatic knee osteoarthritis: a randomized, double-blind, controlled equivalence drug trial. *Rheumatology*.

[35] Soll A. H., Weinstein W. M., Kurata J., McCarthy D. (1991). Nonsteroidal anti-inflammatory drugs and peptic ulcer disease. *Annals of Internal Medicine*.

[36] Xie W., Robertson D. L., Simmons D. L. (1992). Mitogen-inducible prostaglandin G/H synthase: a new target for nonsteroidal antiinflammatory drugs. *Drug Development Research*.

[37] Vane J. R. (1994). Towards a better aspirin. *Nature*.

[38] Frolov R. V., Ignatova I. I., Singh S. (2011). Inhibition of hERG potassium channels by celecoxib and its mechanism. *PLoS ONE*.

[39] ICH Harmonized Tripartite Guideline E14 (2005). *The Clinical Evaluation of QT/QTc Interval Prolongation and Proarrhythmic Potential for Non-Antiarrhythmic Drugs*.

[40] Mukkavilli R., Gundala S. R., Yang C. (2014). Modulation of cytochome P450 metabolism and transport across intestinal epithelial barrier by ginger biophenolics. *PLoS ONE*.

[41] Chen X.-W., Sneed K. B., Pan S.-Y. (2012). Herb-drug interactions and mechanistic and clinical considerations. *Current Drug Metabolism*.

[42] Colombo D., Lunardon L., Bellia G. (2014). Cyclosporine and herbal supplement interactions. *Journal of Toxicology*.

[43] Li M., Chen P. Z., Yue Q. X. (2013). Pungent ginger components modulates human cytochrome P450 enzymes in vitro. *Acta Pharmacologica Sinica*.

[44] Frank A., Unger M. (2006). Analysis of frankincense from various Boswellia species with inhibitory activity on human drug metabolising cytochrome P450 enzymes using liquid chromatography mass spectrometry after automated on-line extraction. *Journal of Chromatography A*.

[45] Rahmani A. H., Al Zohairy M. A., Aly S. M., Khan M. A. (2014). Curcumin: a potential candidate in prevention of cancer via modulation of molecular pathways. *BioMed Research International*.

[46] Gui H.-Y., Chen R.-N., Peng Y. (2013). Curcumin protects against 1-methyl-4-phenylpyridinium ion- and lipopolysaccharide-induced cytotoxicities in the mouse mesencephalic astrocyte via inhibiting the cytochrome P450 2E1. *Evidence-Based Complementary and Alternative Medicine*.

[47] Pimkaew P., Küblbeck J., Petsalo A. (2013). Interactions of sesquiterpenes zederone and germacrone with the human cytochrome P450 system. *Toxicology in Vitro*.

[48] Savai J., Varghese A., Pandita N. (2013). Lack of the cytochrome P450 3A interaction of methanolic extract of *Withania somnifera*, Withaferin A, Withanolide A and Withanoside IV. *Journal of Pharmaceutical Negative Results*.

[49] Rana S., Lattoo S. K., Dhar N. (2013). NADPH-cytochrome P450 reductase: molecular cloning and functional characterizationof two paralogs from *Withania somnifera* (L.) dunal. *PLoS ONE*.

[50] Gabriel S. E., Jaakkimainen L., Bombardier C. (1991). Risk for serious gastrointestinal complications related to use of nonsteroidal anti-inflammatory drugs: a meta-analysis. *Annals of Internal Medicine*.

